# TreeTime: Maximum-likelihood phylodynamic analysis

**DOI:** 10.1093/ve/vex042

**Published:** 2018-01-08

**Authors:** Pavel Sagulenko, Vadim Puller, Richard A Neher

**Affiliations:** 1 Max Planck Institute for Developmental Biology, Spemannstrasse 35, Tübingen 72076, Germany; 2 Biozentrum, University of Basel, Klingelbergstrasse 50, 4056 Basel, Switzerland; 3 SIB Swiss Institute of Bioinformatics, Klingelbergstrasse 50, 4056 Basel, Switzerland

**Keywords:** molecular clock phylogenies, phylodynamics, python

## Abstract

Mutations that accumulate in the genome of cells or viruses can be used to infer their evolutionary history. In the case of rapidly evolving organisms, genomes can reveal their detailed spatiotemporal spread. Such phylodynamic analyses are particularly useful to understand the epidemiology of rapidly evolving viral pathogens. As the number of genome sequences available for different pathogens has increased dramatically over the last years, phylodynamic analysis with traditional methods becomes challenging as these methods scale poorly with growing datasets. Here, we present TreeTime, a Python-based framework for phylodynamic analysis using an approximate Maximum Likelihood approach. TreeTime can estimate ancestral states, infer evolution models, reroot trees to maximize temporal signals, estimate molecular clock phylogenies and population size histories. The runtime of TreeTime scales linearly with dataset size.

## 1. Introduction

Phylogenetics uses differences between homologous sequences to infer the history of the sample and learn about the evolutionary processes that gave rise to the observed diversity. In absence of recombination, this history is a tree along which sequences descend from ancestors with modification. In general, the reconstruction of phylogenetic trees is a computationally difficult problem but efficient heuristics often produce reliable reconstructions in polynomial time (Felsenstein, 2004; Price, Dehal, and Arkin, 2010; Stamatakis, 2014). Such heuristics become indispensable for large datasets of hundreds or thousands of sequences.

Beyond phylogenetic tree building, many research questions require parameter inference and hypothesis testing (Pond and Muse, 2005; Drummond et al. 2012). Again, exact inference from large datasets is computationally expensive since it requires high-dimensional optimization of complex likelihood functions or extensive sampling of the posterior distribution. Efficient heuristics are needed to cope with the growing datasets available today.

One particularly common inference problem is estimating the time of historical events from sequence data. This problem goes back to Zuckerkandl and Pauling (1965), who hypothesized that changes in protein sequences accumulate at a constant rate and that the number of differences between homologous sequences can be used as a ‘molecular clock’ to date the divergence between sequences. Molecular clock methods have since been used to date the divergence of ancient proteins billions of years ago as well as the spread of RNA viruses on time scales less than a year (Langley and Fitch, 1974; Rambaut, 2000; Yoder and Yang, 2000; Sanderson, 2003). Beyond dating of individual divergence events or a common ancestor algorithms have been developed to infer trees where branch lengths correspond directly to elapsed time and each node is placed such that its position reflects its known or inferred date. Such trees are known as time trees, molecular clock phylogenies, or time stamped phylogenies. These methods have been generalized to allow for variation in substitution rates between different branches of the tree and between sites along a sequence. For a recent review of such methods, see (Kumar and Hedges, 2016).

In addition to questions regarding natural history, time trees are useful to study epidemiology and pathogen evolution (Gardy, Loman, and Rambaut, 2015). Time trees of ‘measurably evolving’ pathogens can be used to date cross-species transmissions, introductions into geographic regions, and the time course of pathogen population sizes. In outbreak scenarios such as the recent Ebola virus (EBOV) or Zika virus outbreaks, rapid near real-time analysis of large numbers of viral genomes has the potential to assist epidemiological analysis and containment efforts –provided sample collection, sequencing, and analysis are sufficiently rapid (Gardy, Loman, and Rambaut, 2015).

BEAST is one of most sophisticated tools for time tree estimation (Drummond et al. 2012). BEAST samples many possible histories to evaluate posterior distributions of divergence times, evolutionary rates, and many other parameters. BEAST implements a large number of different phylogenetic and phylogeographic models. The sampling of trees, however, results in run-times of days to weeks for moderately large datasets of a few hundred sequences. On the other end of the spectrum are much simpler distance based tools that infer time scaled phylogenies orders of magnitudes faster (Britton et al. 2007; Tamura et al. 2012; To et al. 2016; Volz and Frost, 2017).

We developed a new tool called TreeTime that combines efficient heuristics with probabilistic sequence evolution models. TreeTime infers maximum likelihood time trees of a few thousand tips within a few minutes. TreeTime was designed for applications in molecular epidemiology and analysis of rapidly evolving heterochronous viral sequences (Volz, Koelle, and Bedford, 2013). It is already in use as an integral component of the real-time time outbreak tracking tools nextstrain and nextflu (Neher and Bedford, 2015). The main applications of TreeTime are ancestral state inference, evolutionary model inference, and time tree estimation. We discuss the core algorithms briefly below.

## 2. Algorithms and implementation

TreeTime’s overarching strategy is to find an approximate maximum-likelihood configuration by iterative optimization of simpler subproblems similar in spirit to ‘sequential quadratic programming’ or ‘expectation maximization’. Iteration is used on multiple levels, for example by iterating optimization of branch lengths, ancestral sequences, parameters of the relaxed clock, or coalescent models. Such an iterative procedure typically converges quickly when the branch lengths of the tree are short such that ancestral sequence inference has little ambiguity.

Ancestral sequences or node positions can be determined to optimize the joint or marginal likelihood. A joint maximum-likelihood assignment corresponds to the global configuration with highest likelihood. In a marginal maximum-likelihood assignment, individual parameters are assigned to the most likely value after summing or integrating over all other unknown states. On a tree, both of these optimal assignments can be calculated in linear time (Pupko et al. 2000; Felsenstein, 2004) and TreeTime implements both marginal and joint ancestral reconstructions for ancestral sequences and node dates.

### 2.1 Iterative branch length optimization

In general, optimizing the branch lengths of a tree is a complicated computational problem with 2*N*−3 free parameters and a likelihood function that requires O(N) steps to evaluate. However, when branch lengths are short and only a minority of sites change on a given branch, a joint optimization of branch lengths and ancestral sequences can be achieved by iteratively inferring branch length and ancestral sequences since corrections due to recurrent substitutions are neglibile. Given a tree topology and the branch length, the maximum-likelihood ancestral sequences can be inferred in linear time (Felsenstein, 2004; Pupko et al. 2000). Likewise maximum-likelihood branch length given the parent and offspring sequences are easy to optimize. We use this iterative optimization scheme to rapidly optimize branch length and ancestral sequences. For more divergent sequences, however, subleading states of internal nodes make a substantial contribution and the iterative optimization will underestimate the branch lengths. In this case, TreeTime can use branch lengths provided in the input tree.

### 2.2 Maximum-likelihood inference of divergence times

For a fixed tree topology, TreeTime infers ancestral sequences maximizing the joint sequence likelihood (see above). The branch lengths corresponding to the maximum-likelihood molecular clock phylogeny can be computed in linear time using dynamic programming or message passing techniques (Mézard and Montanari, 2009). This approach is similar to the approach by Rambaut (2000), but the dynamic programming technique avoids computationally expensive numerical optimization of the branch lengths.

**Figure 1.**
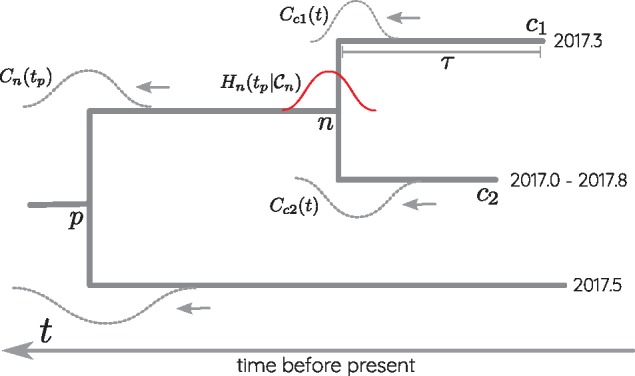
Illustration of TreeTime’s time tree inference algorithm. Terminal nodes in the tree are either associated with exact dates or date ranges (node *c*_2_ in this example). These temporal constraints are convolved with the distribution $b_{c_{i}}(\tau )$ of the branch length *τ* leading to node *c_i_* to yield $C_{c_{i}}(t)$. At the internal node *n*, the messages from children *c*_1_ and *c*_2_ are multiplied and contribute to $H_{n}(t|C_{n})$. The latter is further passed down to the parent by convolving with $b_{n}(\tau )$.

In analogy to maximum-likelihood inference of ancestral sequences the algorithm proceeds via a post-order tree traversal propagating the maximum-likelihood assignments of subtrees towards the root, and a pre-order traversal selecting the optimal subtree given the placement of the parent node. Specifically, we calculate in post-order for each node *n*

(1) $$H_{n}(t|C_{n})=E_{n}(t)\prod_{c\in C_{n}}C_{c}(t)\;,$$

the likelihood that the node sits at position *t* given the information and constraints propagated from its children $C_{n}$. $E_{n}(t)$ accounts for external contraints imposed on the date of the node (e.g. fossil dating), while the product runs over all children *c* of node *n* and multiplies the integrated messages of all subtending nodes. The time *t* is measured as time before present. Temporal information is propagated along the branches of the tree via

(2) $$C_{n}(t_{p})=\underset{\tau }{\max\;}b_{n}(\tau ){\text{H}}_{n}(t_{p}-\tau |C_{n})\;,$$

where $b_{n}(\tau )$ is the probability distribution of the branch length *τ* between the focal node *n* and its parent. This distribution is conditional on the sequences assigned to node *n* and its parent. Intuitively, $C_{n}(t_{p})$ specifies the distribution of the date *t_p_* of the parent of node *n*, given the constraints from the tips descending from node *n* and the substitutions that accumulated on the branch to the parent node. The different objects are illustrated in Fig. 1.

During the post-order traversal, the branch lengths $\tau (t_{p})$ maximizing Equation (2) for a given *t_p_* are tabulated and saved for the back-trace. Once the post-order traversal arrives at the root, the root is assigned the time $t_{n}={\text{argmax}}_{t}E(t)\prod_{c}C_{c}(t)$.

The post-order traversal is followed by a pre-order back-trace during which the branch length of each internal node is assigned to the optimal $\tau (t_{p})$ conditional on the parental position *t*_p_. To accelerate the optimization, TreeTime tabulates the branch length likelihood function $b_{n}(\tau )$ and the subtree origin likelihoods $H_{n}(t|C_{n})$.

The above algorithm assigns each node to the time that maximizes the joint likelihood of all branch lengths in analogy to the ancestral state reconstruction algorithm by Pupko et al. (2000). The marginally optimal time of each internal node, that is, the time after integration over all other unconstrained nodes, can be determined in a similar manner by replacing the max in Equation (2) by a convolution integral over *τ*

(3) $$C\prime_{n}(t_{p})=\int_{0}^{\infty }b_{n}(\tau )H\prime_{n}(t_{p}-\tau |C_{n})\;d\tau \;,$$

where $H\prime_{n}(t|C_{n})$ is the analog of $H_{n}(t|C_{n})$ in Equation (1) multiplying the $C\prime_{c}$ of all children and any external date prior.

Once the post-order transversal arrives at the root, the marginal distribution of time *t* of the root node *r* is given by

(4) $$P_{r}(t)=\frac{E_{r}(t)}{Z_{r}}\prod_{c\in C_{r}}C_{c}^{\prime }(t)$$

where *Z_r_* is a normalization factor. The corresponding marginal distributions of other nodes are then calculated during a pre-order traversal via

(5) $$P_{n}(t)=\frac{1}{Z_{n}}H_{n}(t|C_{n})\int_{0}^{\infty }b(\tau )\frac{P_{p}(t+\tau )}{C\prime_{n}(t+\tau )}\;d\tau \;.$$

The factor $H_{n}(t|C_{n})$ accounts for the date information coming from the leaves of node *n*, while the integral contributes the date information from clades other than node *n* and its children. Note that the contribution of node *n* to *P_p_* is removed by dividing $P_{p}(t+\tau )$ by $C_{n}^{\prime }(t+\tau )$.

The result of the marginal reconstruction is a probability distribution of the node date given the tree, the ancestral sequence assignment, and the evolutionary model while the unknown times of other nodes are traced out. From this distribution, confidence intervals of node dates can be computed in a straight-forward manner.

TreeTime allows one to compute joint or marginal maximum-likelihood dates, but the algorithm described above can be used for any continuous character on the tree. In Equation (2), $b_{n}(\tau )$ can be replaced by any transmission function that depends either on the branch or properties of the child and parent node. We will use an analogous algorithm below to estimate parameters of relaxed molecular clock models.

### 2.3 Efficient search for the optimal root

The fraction of variance in root-to-tip (RTT) distance explained by a linear regression on sampling date is given by

(6) $$r^{2}=\frac{{\left(\sum_{i}(t_{i}-\langle t\rangle )(d_{i}-\langle d\rangle )\right)}^{2}}{\sum_{k}(t_{k}-\langle t\rangle {)}^{2}\sum_{l}(d_{l}-\langle d\rangle {)}^{2}}$$

where the sums run over all tips of the tree and *t_i_* and *d_i_* are the sampling date and the distance from the root to node *i*, respectively. The distances *d_i_* are measured as the sum of lengths of all branches from the root to the tip, that is, the expected number of substitutions since the root divided by the length of the sequence. The angular brackets denote the sample average. The regression and *r*^2^ depend on the choice of root since the *d_i_* depend on the root. In absence of an outgroup, the root is often chosen to maximize *r*^2^ or minimize the squared residuals of a linear fit to the RTT distance. Programs such as TempEst (Rambaut et al. 2016) and LSD (To et al. 2016) allow to search for the root that maximizes this correlation and TreeTime has implemented similar functionality.

This search for the optimal root can be achieved in linear time in the number of sequences *N* by first calculating

(7) $$\theta_{n}=\sum_{i\in L_{n}}d_{n,i}\;,\;\gamma_{n}=\sum_{i\in L_{n}}t_{i}d_{n,i}\;\text{and}\;\delta_{n}=\sum_{i\in L_{n}}d_{n,i}^{2}$$

for each internal node *n*. Here, the sum runs over all tips $i\in L_{n}$ of node *n* while *t_i_* and $d_{n,i}$ are the sampling date and the distance of tip *i* from node *n*, respectively. The quantities *θ_n_*, *γ_n_*, and *δ_n_* can be calculated recursively from *θ_c_*, *γ_c_*, and *δ_c_* of the child nodes in one post-order traversal. Once those quantities are calculated, the corresponding quantities Θ_*n*_, Γ_*n*_, and Δ_*n*_ that sum contributions from all tips—not just the subtending ones—can be calculated in one pre-order traversal.

With these quantities at hand, *r*^2^ can be calculated for any choice of root on the tree as detailed in the Appendix. Hence two tree traversals are sufficient to determine the optimal root. The root position that minimizes the mean squared residual can be calculated analogously.

In general, the optimal position of the root will not be an internal node, but a position between two nodes on a branch of the tree. Such optimal position on internal branches of the tree can be determined from the quantities calculated above by solving a quadratic equation without any numerical optimization. The required algebra is described in the Appendix.

### 2.4 Resolving polytomies

Phylogenetic trees of many very similar sequences are often poorly resolved and contain multifurcating nodes also known as polytomies. Tree building software often randomly resolves these polytomies into a series of bifurcations. However, the order of bifurcations will often be inconsistent with the temporal structure of the tree resulting in poor approximations. To overcome this problem, TreeTime can prune all branches of length zero and resolve the resulting polytomies in a manner consistent with the sampling dates. For each pair of nodes, TreeTime calculates by how much the likelihood would increase when grouping this pair of nodes into a clade of size two. The polytomy is then resolved iteratively by always grouping pairs corresponding to the highest gain.

### 2.5 Coalescent models

The likelihood of observing a particular genealogical tree depends on the size of the population, its geographic structure, and fitness variation in the population (Kingman, 1982; Nordborg, 1997; Neher, 2013). Hence parameters of models describing the ensemble of genealogies can be estimated from the data.

In the simplest case of a panmictic population without fitness variation, the ensemble of genealogies is described by a Kingman (1982) coalescent, possibly with a population size that changes over time. Within the Kingman coalescent, merger events occur at random with a rate λ(t) that depends on the population size *N*(*t*) and the current number of lineages *k*(*t*).

(8) $$\lambda (t)=\frac{k(t)(k(t)-1)}{2N(t)}$$

Here, the population size *N*(*t*) defines a time scale measured in units of generation time and we will more generally refer to this time scale by $T_{c}(t)$ and measure it in units of the inverse clock rate.

The contribution of a branch between time points *t*_0_ (child) and *t*_1_ (parent) in the tree to the likelihood is then given by

(9) $$p(t_{0},t_{1})=e^{-\int_{t_{0}}^{t_{1}}\kappa (t)\;dt}\;,$$

where $\kappa (t)=(k(t)-1)/2T_{c}(t)$ is the rate at which a given lineage merges with any of the other. A merger at time *t* contributes a factor λ(t) to the coalescent likelihood.

TreeTime can estimate population sizes or coalescent time scales by maximizing the likelihood contribution of the coalescent likelihood for a fixed tree. The latter can be evaluated in one tree traversal by summing contributions from branches and merger events. In addition to a constant *T*_c_, TreeTime can model *T*_c_ as a piecewise linear function and optimize the parameters of that function. Such piecewise functions are known as ‘skyline’ (Strimmer and Pybus, 2001).

As part of the iterative optimization by TreeTime, the next round of optimization of branch lengths and dates of ancestral nodes will account for the coalescent likelihood. The newly inferred dates will in turn be used to update the parameters of the coalescent model as described earlier.

### 2.6 Inference of time reversible substitution models

Large phylogenies typically contain 100s of substitutions and thus provide enough information to infer substitution models from the data. General time reversible (GTR) substitution models (Felsenstein, 2004) are parameterized by equilibrium state frequencies *π_i_* and a symmetric substitution matrix *W_ij_*. The substitution rate from state j→i is then $Q_{ij}=\pi_{i}W_{ij}$.

TreeTime infers parameters of GTR models via an iterative procedure similar to Expectation–Maximization algorithms. TreeTime first reconstructs ancestral sequences using a standard substitution model specified by the user (Jukes-Cantor by default). From this reconstruction, TreeTime calculates the time *T_i_* spent in different states *i* across the tree, and the number of substitutions *n_ij_* between any pair of states (*i*, *j*). Then, *π* and *W* are determined by iterating the two equations

(10) $$W_{ij}=\frac{n_{ij}+n_{ji}+2p_{c}}{\pi_{i}T_{j}+\pi_{j}T_{i}+2p_{c}}$$

(11) $$\pi_{i}=\frac{\sum_{j}n_{ij}+p_{c}+m_{i}}{\sum_{j}W_{ij}T_{j}+\sum_{j}\left(m_{j}+p_{c}\right)}\;,$$

where *p_c_* is a small pseudo-count driving the estimate towards a flat Jukes-Cantor model in absence of data, and the *m_i_* are the number times state *i* is observed in the sequence of the root. *W_ij_* are evaluated at fixed *π*, followed by calculating *π* with the current *W_ij_*. After each iteration, *π* is normalized to one, the diagonal of *W_ij_* is set to $-\pi_{i}^{-1}\sum_{j\neq i}W_{ij}\pi_{j}$, and *W_ij_* is rescaled such that the total expected substitution rate $-\sum \pi_{i}W_{ii}\pi_{i}$ equals one. The rescaling of *π* and *W_ij_* can be absorbed into an overall rate *μ*. This algorithm typically converges in a few iterations.

### 2.7 Relaxed clocks

Substitution rates can vary across the tree and models that assume constant clock rates may give inaccurate inferences. Models that allow for clock rate variation have been proposed (Hasegawa, Kishino, and Yano, 1989; Yoder and Yang, 2000; Drummond et al. 2006). These models typically regularize clock rate variation through a prior and penalize rapid changes of the rate by coupling the rate along branches—known as autocorrelated or local molecular clock (Thorne, Kishino, and Painter, 1998; Aris-Brosou, Yang, and Huelsenbeck, 2002).

TreeTime implements an autocorrelated molecular with a normal prior on variation in clock rates. The choice of the normal prior allows for an exact and linear time solution for the maximum-likelihood substitution rates via the same forward/backward trace algorithm used for the inference of dates of internal nodes. Other priors could be implemented, but would require numerical optimization or approximations.

### 2.8. Implementation

TreeTime is implemented in Python (version 2.7) and uses the packages numpy and scipy for optimization, linear algebra, and interpolation Jones et al. (2001–2017) and van der Walt, Colbert, and Varoquaux (2011). Computationally costly operations are cast into array operations executed by numpy whenever possible.

TreeTime is organized as a hierarchy of classes. TreeAnc performs maximum-likelihood inference of ancestral sequences, ClockTree infers a time scaled phylogeny given a tree topology, and TreeTime adds an additional layer of functionality including rerooting, polytomy resolution, coalescent models, and relaxed clocks. The substitution model is implemented in the class GTR.

This structure allows TreeTime to be used in a modular fashion in Python based phylogenetic analysis pipelines. In addition, scripts can be called from the command line to perform standard tasks such as ancestral sequence inference, rerooting of trees, and time tree estimation.

### 2.9 Availability

TreeTime is published under an MIT license and available at github.com/neherlab/treetime. Data and scripts necessary used to validate TreeTime are available at github.com/neherlab/treetime_validation. TreeTime can be used via a web interface at treetime.ch.

## 3. Validation and performance

To assess the accuracy of date reconstructions of TreeTime and to compare its performance to existing tools such as Bayesian Evolutionary Analysis Sampling Trees (BEAST) and Least-square dating (LSD) (Drummond et al. 2012; To et al. 2016), we generated toy data using the FFPopSim forward simulation library (Zanini and Neher, 2012). We simulated populations of size *N* = 100 and used a range evolutionary rates $\mu =[10^{-5},\ldots ,2\cdot 10^{-3}]$ resulting in expected genetic diversity from 0.001 to 0.2. Sequences were sampled every 10, 20, or 50 generations. The length of the simulated sequences was *L* = 1000.

**Figure 2.**
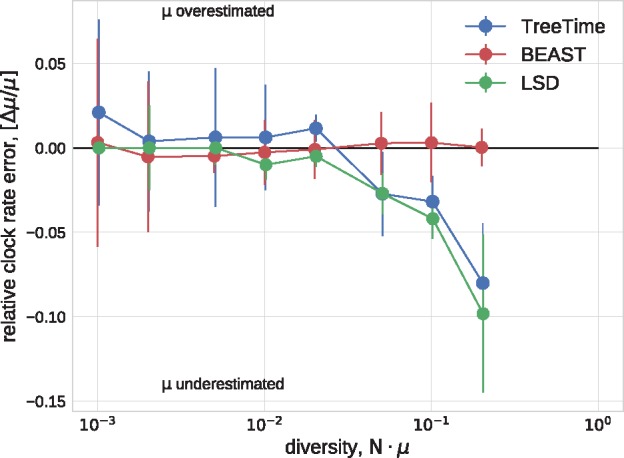
Estimation of the evolutionary rate from simulated data. TreeTime and LSD (following tree reconstruction with FastTree) underestimated the rate when branch lengths are long but return accurate estimates for low diversity samples. The graph shows median values, error bars indicate the inter-quartile distances.

Fig. 2 shows the error in the estimates of the clock rate for TreeTime, LSD, and BEAST as a function of genetic diversity. TreeTime and LSD estimated the clock rate accurately at low diversity but tended to underestimate the rates at when diversity exceeds a few percent. This is expected in the case of TreeTime since maximum-likelihood sequence assignment can result in underestimated branch lengths. BEAST produced accurate estimates across the entire range of diversities. By sampling trees, BEAST does not suffer from the atypical maximum-likelihood assignments.

**Figure 3.**
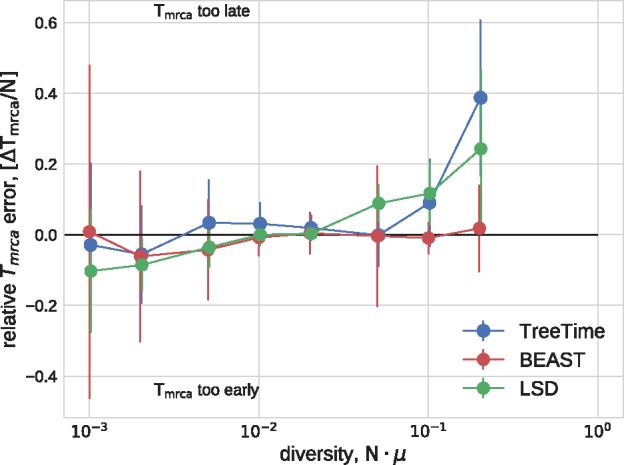
Estimation of the *T_MRCA_* from simulated data. TreeTime, LSD, and BEAST estimated the time of the MRCA within 10% accuracy at low diversity, but TreeTime and LSD tended to overestimate the date of the root when branch lengths are long. The graph shows median values, error bars indicate the inter-quartile distances.

In a similar manner, TreeTime and LSD estimated the time of the most recent common ancestor to within 10% accuracy at low diversity (relative to the coalescence time) with larger deviations at diversity above 10%, see Fig. 3. BEAST returned accurate estimates across the entire range of diversities.

**Figure 4.**
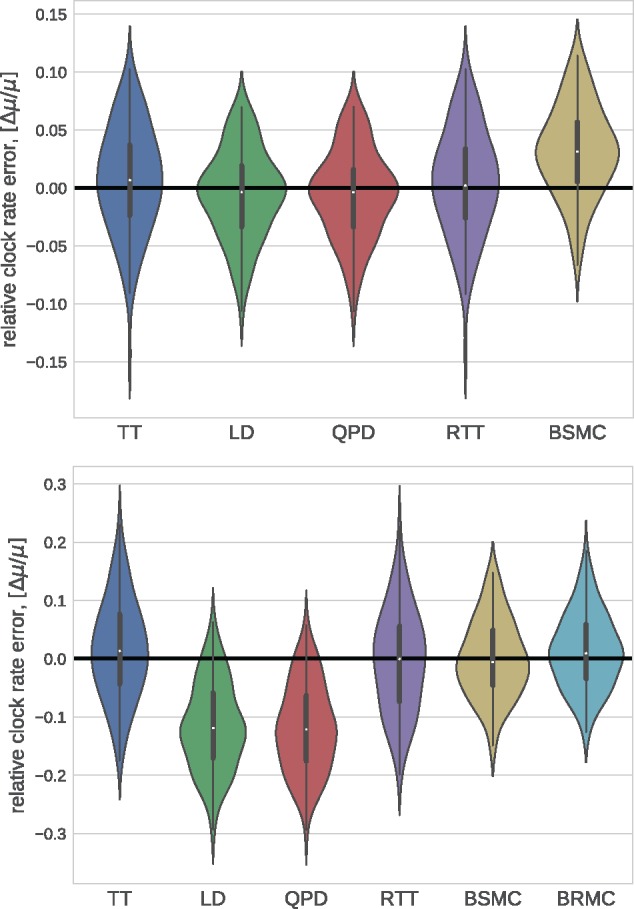
Method comparison on LSD test data. TreeTime (TT) showed comparable or better accuracy as BEAST (strict clock: BSMC; relaxed clock: BRMC), LSD (linear dating: LD; quadratic programming dating: QPD), or RTT regression when run on simulated data provided by (To *et al.*, 2016). Both panels use the tree set 750_11_10, the top and bottom panel show runs on alignments generated with a strict and relaxed molecular clock, respectively.

We also ran TreeTime on simulated data provided by To et al. (2016) and compared it to the results reported by To et al. (2016) for LSD, BEAST, and a number of other methods. Figure 4 compares the accuracy of *T*_MRCA_ and clock rate estimates, showing that TreeTime achieves similar or better accuracy than other methods.

### 3.1 Coalescent model inference

**Figure 5.**
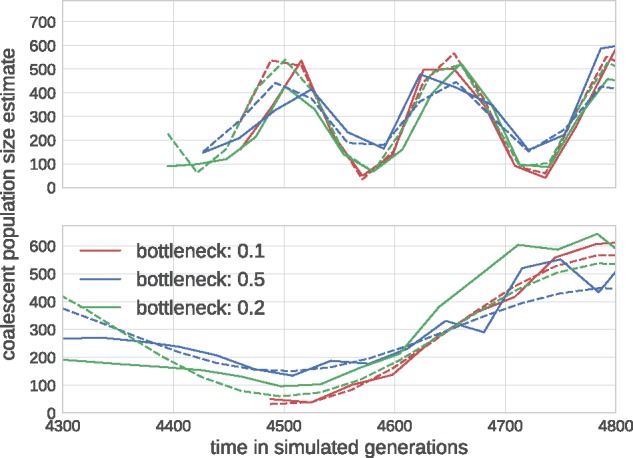
Reconstruction of fluctuating population sizes by TreeTime. The graph shows simulated population size trajectories (dashed lines) and the inference by TreeTime as solid lines of the same color. Different lines vary in the bottleneck sizes of 10% (red), 20% (green), and 50% (blue) of the average population size. The top panel shows data for fluctuations with period 0.5 *N*, the bottom panel 2 *N*. The average population size is *N* = 300.

Population bottlenecks, selective sweeps, or population structure affect the rate of coalescence in a time-dependent manner. BEAST can infer a history of effective population size (inverse coalescence rate) from a tree—often known as skyline. TreeTime can perform a similar inference by maximizing the coalescence likelihood with respect to the pivots of a piecewise linear approximation of the coalescence rate history *T*_c_(*t*) (aka effective population size). To test the power and accuracy of this inference, we simulated sinusoidal population size histories of different amplitude and period, uniformly sampled sequences through time, and used these data to estimate the coalescent rate history. True and estimated population size histories agree well with each other as shown in Fig. 5.

### 3.2 Influenza phylogenies

**Figure 6.**
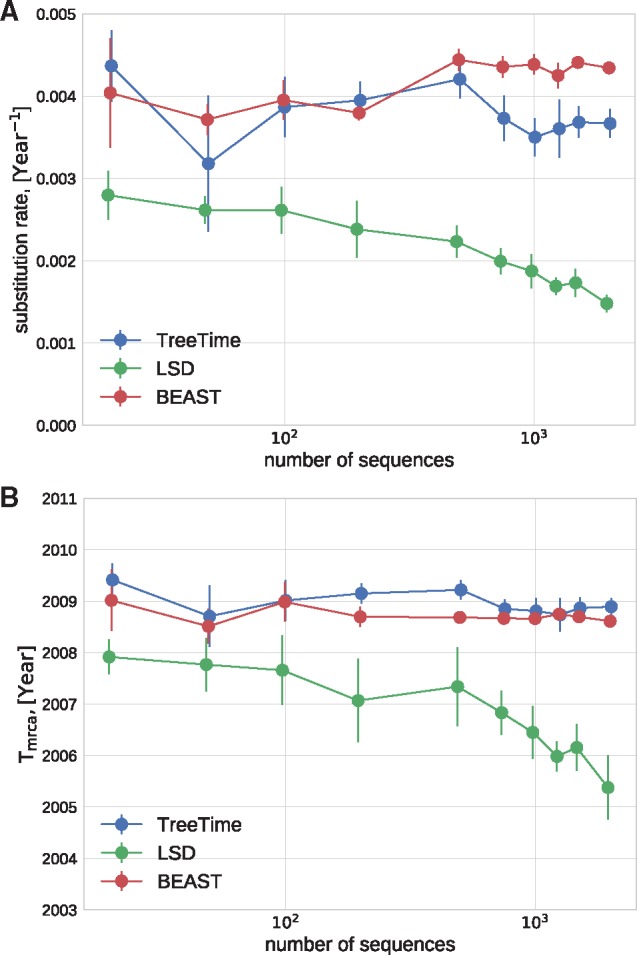
Sensitivity the dataset size. TreeTime and BEAST returned consistent estimates of the rate of evolution (A) and the *T*_MRCA_ (B) when analyzing alignments of Influenza A/H3N2 HA sequences of various size. LSD showed a systematic drift.

The dense sampling of influenza A virus sequences over many decades makes this virus an ideal test case to evaluate the sensitivity of time tree estimation to sampling depth. We estimated the clock rate and the time of the most recent common ancestor of influenza A/H3N2 HA sequences sampled from 2011 to 2013 for sets of sequences varying from 30 to 3,000, see Fig. 6. TreeTime estimates are stable across this range, while estimates by LSD tend to drift with lower rates and older MRCAs for larger samples. Estimates by BEAST are generally consistent with TreeTime.

Next, we tested how accurately TreeTime infered dates of tips when only a fraction of tips have dates assigned. Every tip in TreeTime can either be assigned a precise date, an interval within which the date is assumed to be uniformly distributed, or no constraint at all. TreeTime will then determine the probability distribution of the date of the node based on the distribution of the ancestor and the substitutions that occurred since the ancestor.

**Figure 7.**
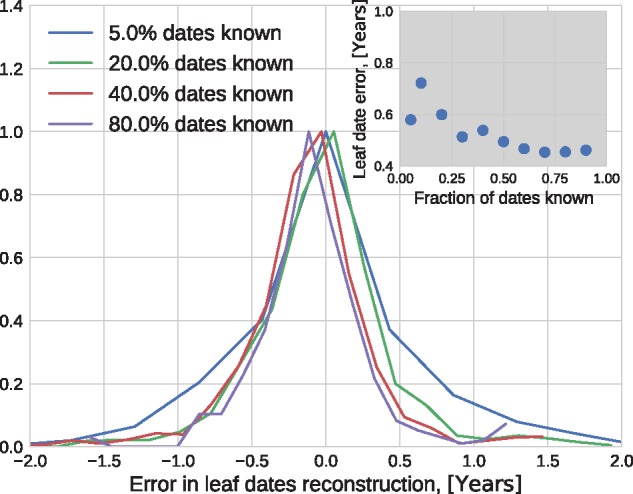
Sensitivity to missing information. The inter-quartile range of the error of estimated tip dates decreases from 0.7 to 0.5 years as the fraction of known dates increases from 5 to 90% (see inset).

Figure 7 shows the distribution of error in leaf date reconstruction as the fraction of missing dates increased from 5 to 95% of all nodes. TreeTime estimated the date of influenza sequences to an average accuracy of ≈0.5 years if >50% of dates are known.

### 3.3 Analysis of the 2014–15 EBOV outbreak

**Figure 8.**
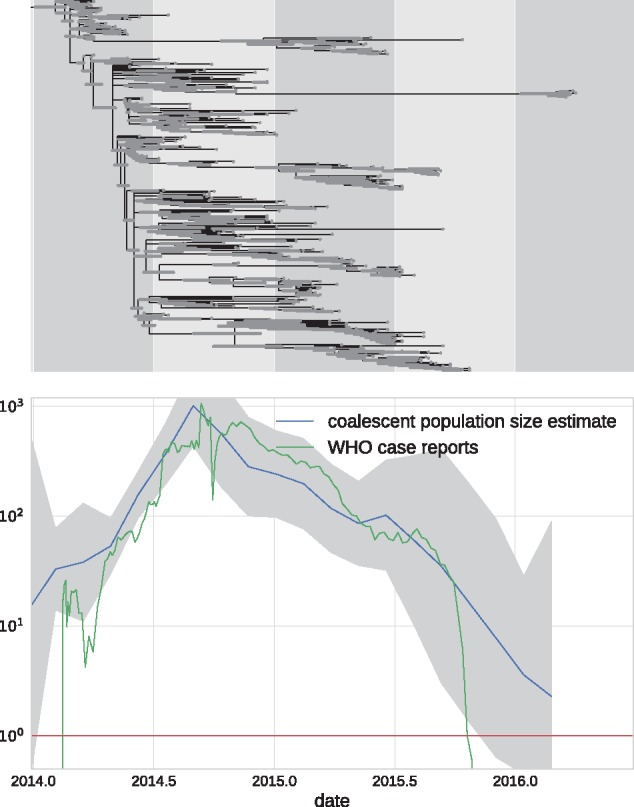
EBOV phylodynamic analysis. The top panel shows a molecular clock phylogeny of EBOV sequences obtained over from 2014 to 2016 in West Africa. The lower panel shows the estimate of the coalescent population size along with its confidence intervals. The estimate suggests an exponential increase until late 2014 followed by a gradual decrease leading to almost complete eradication by 2016. Ebola case counts, as reported by the WHO (2016) agree quantitatively with the estimate.

In 2014, West Africa experienced the largest known outbreak of EBOV in humans. The genomic epidemiology has been studied intensively by multiple groups (Dudas et al. 2017). Here, we reanalyzed a subset of 350 EBOV sequences sampled throughout the outbreak from 2014–16. Due to the dense sampling, the maximum-likelihood phylogeny has many unresolved nodes and TreeTime was used to resolve polytomies using temporal information. After automatic rooting and GTR model inference, TreeTime produced the time tree shown in Fig. 8. The GTR model inferred from the tree was

(12) $$\pi =\begin{array}{cc} A: & 0.32\\ C: & 0.21\\ G: & 0.195\\ T: & 0.275 \end{array}\;W=\begin{array}{ccccc} & A & C & G & T\\ A & \cdot & 0.45 & 2.7 & 0.28\\ C & 0.45 & \cdot & 0.25 & 3.7\\ G & 2.7 & 0.25 & \cdot & 0.45\\ T & 0.28 & 3.7 & 0.45 & \cdot \end{array}$$

This analysis took 4 min to complete on a 2016 laptop (Dell XPS13) with an i7 processor using a single CPU. In addition to inferring a time tree, TreeTime estimated the time course of the coalescent population size shown in the lower panel of Fig. 8. The estimated population size closely mirrors the case counts reported by the WHO throughout this period.

## 4. Discussion

TreeTime was developed to analyse large heterochronous viral sequence alignments and we have used TreeTime as the core component of the real-time phylogenetics pipelines nextstrain and nextflu (Neher and Bedford, 2015). TreeTime tries to strike a useful compromise between inflexible but fast heuristics and computationally expensive Bayesian approaches that require extensive sampling of treespace. The overarching algorithmic strategy is iterative optimization of efficiently solvable subproblems to arrive at a consistent approximation of the global optimum. Although this strategy is approximate and often assumes short branch length, it converges fast for many applications and trees with thousands of tips can be analyzed in a few minutes. In this paper, we presented analyses of human seasonal influenza A/H3N2 virus sequences and sequences of the recent EBOV outbreak. In both cases, average pairwise distances between strains are 10% and individual branches in the trees are much shorter still. TreeTime assumption of short branches is therefore met.

Rapid, efficient analysis phylodynamic algorithms are of increasing importance as datasets are increasing in size. For example during the recent outbreaks of EBOV and Zika virus, hundreds of sequences were generated and needed to be analyzed in near real time to inform containment efforts. Similarly, the GISRS network for surveillance of seasonal influenza virus sequences hundreds of viral genomes per month. Timely analysis of these data with Bayesian methods that require extensive tree sampling such as BEAST is difficult. Sequencing from EBOV, Zika virus outbreaks, or seasonal influenza viruses are typically very similar to each other (>90% identity) such that TreeTime assumptions and approximations are justified.

When compared with other methods recently developed for rapid estimation of time trees (Britton et al. 2007; Tamura et al. 2012; To et al. 2016), TreeTime uses probabilistic models of evolution, allows inference of ancestral characters, and coalescent models. In TreeTime, every node of the tree can be given a strict or probabilistic date constraint. This higher model complexity results in longer run times, but the scaling of run times remains linear in the size of the dataset and alignments with thousands of sequences can be analyzed routinely. The time tree inference and dating are typically faster than the estimation of the tree topology.

TreeTime was tested predominantly on sequences from viruses with a pairwise identity above 90%. The iterative optimization procedures are not expected to be accurate for trees were many sites are saturated. In scenarios with extensive uncertainty of ancestral states and tree topology, convergence of the iterative steps cannot be guaranteed. While in many cases TreeTime might still give approximate branch lengths, ancestral assignments and time tree estimates, these need to be checked for plausibility. In general global optimization and sampling of the posterior can not be avoided.

TreeTime can be used in a number of different ways. The core TreeTime algorithms and classes can be used in larger phylogenetic analysis pipelines as Python scripts. This is the most flexible way to use TreeTime and all the different analysis steps can be combined in custom ways with user specified parameters. In addition, we provide command-line scripts for typical recurring tasks such as ancestral state reconstruction, rerooting to maximize temporal order, and time tree inference. We also implemented a web-server that allows exploration and analysis of heterochronous alignments in the browser without the need to use the command-line.

**Conflict of interest:** None declared.

## Appendix

To calculate the correlation between the RTT distances and tip dates via Equation (6), one first needs to calculate the means and (co)variances of tip dates and RTT distances. For a tree with *N* tips, this requires O(N) operations and calculating it for all internal nodes would therefore require $O(N^{2})$ operations. The same covariances are needed to calculate the regression parameters and the residuals. However, its is possible to calculate the quantities for all nodes at once, reducing the total number of operations to O(N).

The speed-up is possible through recursively calculating sums and averages on the tree. We denote the set of tips that descend of node *n* by $L_{n}$. We will need the number tips $M_{n}=|\ell_{n}|$, the sum of their sampling times $\tau_{n}=\sum_{i\in L_{n}}t$, the sum of their distances $d_{n,i}$ from node *n*$\theta_{n}=\sum_{i\in L_{n}}d_{n,i}$, and the analogous higher order quantities $\gamma_{n}=\sum_{i\in L_{n}}t_{i}d_{n,i}$ and $\delta_{n}=\sum_{i\in L_{n}}d_{n,i}^{2}$.

First, assign *M_n_* = 1, $\tau_{n}=t_{n},\;\theta_{n}=0,\;\gamma_{n}=0$ and $\delta_{n}=0$ for all tips of the tree. Then, in one post-order transversal over internal nodes, we can calculate these quantities by summing the following expressions over the children $C_{n}$ of node *n*.

(13) $$M_{n}=\sum_{c\in C_{n}}M_{c}$$

(14) $$\begin{array}{l} \tau_{n}=\sum_{c\in C_{n}}\tau_{c}\\ \theta_{n}=\sum_{c\in C_{n}}M_{c}l_{c}+\theta_{c}\\ \gamma_{n}=\sum_{c\in C_{n}}l_{c}\tau_{c}+\gamma_{c}\\ \delta_{n}=\sum_{c\in C_{n}}M_{n}l_{c}^{2}+2l_{c}\theta_{n}+\delta_{c} \end{array}$$

The length of branches leading from node *n* to child *c* is denoted by *l_c_*. To calculate the covariances at a particular node *n*, we need to sum over all terminal nodes rather than only tips that descend from the node. We denote the corresponding quantities by capital letters. The sums of the sampling dates and their squares are of course straightforward to evalulate, the remaining quantities that depend on the choice of the focal node *n* can be calculated in one pre-order transversal. Let *p* denote the parent node of node *n*

(15) $$\begin{array}{l} \Theta_{n}=\Theta_{p}-(N-2M_{n})l_{n}\\ \Gamma_{n}=\Gamma_{p}+l_{n}(T-2\tau_{n})\\ \Delta_{n}=\Delta_{p}+2l_{n}\Theta_{p}-4l_{n}(\theta_{n}+(N-M_{n})l_{n})+Nl_{n}^{2} \end{array}$$

Note that the order in which these calculations are performed matters. The first line, calculating Θ_*n*_ adjusts the parent value Θ_*p*_ for the fact that the branch leading to node *n* is transversed by $N-M_{n}$ path instead of *M_n_* if the root is shifted from *p* to *n*. Similarly, Γ_*n*_ is calculated from Γ_*p*_ by adjusting with the difference of sum of times of subtending and complementary nodes. The corresponding expression for the sum of squared RTT distances is slightly more complicated but still follows from elementary algebra.

With these quantities at hand, the regression, residuals, and *r*^2^ can be straightforwardly calculated from the means and covariances given by

(16) $$\begin{array}{l} \langle d_{n,i}\rangle =\frac{\Theta_{n}}{N}\\ \langle d_{n,i}t_{i}\rangle -\langle d_{n,i}\rangle \langle t_{i}\rangle =\frac{\Gamma_{n}}{N}-\frac{\Theta_{n}T}{N^{2}}\\ \langle d_{n,i}^{2}\rangle -{\langle d_{n,i}\rangle }^{2}=\frac{\Delta_{n}}{N}-\frac{\Theta_{n}}{N^{2}} \end{array}$$

In general, the optimal root is not going to coincide with a preexisting node but will be placed somewhere along a branch. When placing the root at a position ϵ∈[0,1] along the branch, the corresponding $\Theta_{n}(\epsilon ),\;\Gamma_{n}(\epsilon ),\;\Delta_{n}(\epsilon )$ are obtained by substitution $\epsilon l_{c}$ for *l_c_* in Equation (15). The fraction of variance explained by a RTT regression with a root placed at position ϵ on a branch then has the generic form

(17) $$r^{2}=\frac{{\left(a+b\epsilon \right)}^{2}}{r+s\epsilon +t\epsilon^{2}}$$

where the coefficients a,b,r,s and *r* can be obtained by substituting the expressions for $\Theta_{n}(\epsilon ),\;\Gamma_{n}(\epsilon )$, and $\Delta_{n}(\epsilon )$. The term a+bϵ, for example, evaluates to

(18) $$\begin{array}{cc} a+b\epsilon = & \langle d_{n,i}t_{i}\rangle -\langle d_{n,i}\rangle \langle t_{i}\rangle \\ = & \frac{\Gamma_{n}(\epsilon )}{N}-\frac{\Theta_{n}(\epsilon )T}{N^{2}}\;. \end{array}$$

Substituting $\Theta_{n}(\epsilon )$ and $\Gamma_{n}(\epsilon )$ and collecting terms by powers of ϵ, the coefficients *a* and *b* can be read off. The condition for a maximum $\frac{dr^{2}}{d\epsilon }=0$ results in an quadratic equation for ϵ. Hence, the optimal position of the root can be calculated with a number of operations that increases linearly in the size of the tree.

The slope of the RTT regression or clock rate in then simply $\alpha =\frac{\langle d_{i}t_{i}\rangle -\langle d_{i}\rangle \langle t_{i}\rangle }{\langle t_{i}^{2}\rangle -{\langle t_{i}\rangle }^{2}}$, where *d_i_* are evaluated with respect to the optimal root.
